# Operational Research to Inform Programmatic Approaches to the Management of Tuberculosis in Uzbekistan

**DOI:** 10.3390/ijerph182312308

**Published:** 2021-11-23

**Authors:** Jamshid Gadoev, Anthony D. Harries, Oleksandr Korotych, Ajay M. V. Kumar, Andrei Dadu, Lianne Kuppens, Nargiza Parpieva, Barno Abdusamatova, Askar Yedilbayev, Masoud Dara

**Affiliations:** 1 World Health Organization Country Office in Uzbekistan, Tashkent 100100, Uzbekistan; kuppensl@who.int; 2 International Union against Tuberculosis and Lung Disease, 75006 Paris, France; adharries@theunion.org (A.D.H.); akumar@theunion.org (A.M.V.K.); 3 London School of Hygiene and Tropical Medicine, Keppel Street, London WC1E 7HT, UK; 4 World Health Organization Regional Office for Europe, DK-2100 Copenhagen, Denmark; korotycho@who.int (O.K.); dadua@who.int (A.D.); yedilbayeva@who.int (A.Y.); daram@who.int (M.D.); 5 International Union against Tuberculosis and Lung Disease, South-East Asia Office, New Delhi 110016, India; 6 Yenepoya Medical College, Yenepoya (Deemed to be University), Mangaluru 575018, India; 7 Republican Specialized Scientific-Practical Medical Center Phthisiology and Pulmonology, Tashkent 100086, Uzbekistan; nargizaparpieva@gmail.com; 8 Ministry of Health of Republic of Uzbekistan, Main Department of Protection of Maternity and Childhood, Tashkent 100011, Uzbekistan; barno.abdusamatova@minzdrav.uz

**Keywords:** tuberculosis, drug-resistant tuberculosis, operational research, Uzbekistan, SORT-TB, SORT IT

## 1. Progress towards the Goal of Ending TB by 2030

Globally, an estimated 10 million people fell ill with tuberculosis (TB) in 2019, a number that has been declining very slowly in recent years. According to the latest global TB report, the reduction in global TB incidence is too slow and the global 2020 milestone of a 20% decrease compared with 2015 will not be met. The cumulative reduction of TB incidence between 2015 and 2019 was only 9% worldwide. A similar trend has been observed with respect to TB mortality: the number of TB deaths is falling globally and the cumulative reduction between 2015 and 2019 reached 14%, which is far below the global milestone for 2020 (35% reduction compared with 2015). The only region which is on track to meet the 2020 milestones of the End TB Strategy is the World Health Organization (WHO) European Region, with cumulative decreases in TB incidence of 19% and TB mortality of 31% as of 2019 [1]. Despite the progress achieved, TB remains a public health challenge in the WHO European Region, specifically in eastern European and central Asian countries [2].

The WHO End TB Strategy serves as a blueprint for countries to reduce TB incidence by 80% and TB deaths by 90% and to eliminate catastrophic costs for TB-affected households by 2030 [3]. Research is critical to bend the trajectory of the epidemic and reach the global targets to end TB [4]. Intensified research and innovation comprise the third pillar of the End TB Strategy. This is needed to increase the effectiveness of existing tools and develop innovative technologies to transform the way TB is diagnosed, treated and prevented. The WHO has developed a Global Action Framework for TB Research which aims to foster high-quality national and global TB research over the next years [5]. The Framework has two fundamental objectives: (i) to promote, enhance and intensify TB research and innovation at the country level, with a focus on low- and middle-income countries, through the development of country-specific TB research plans and strong research capacity to drive this work: to support this objective, WHO has launched a “Toolkit for Developing and Implementing a National TB Research Plan” [6]; and (ii) to promote, enhance and catalyze TB research at the global level through advocacy, sharing innovations, discussing global priorities in TB research and developing regional and international networks for research and capacity building.

More recently, the Global Strategy for TB Research and Innovation was adopted by all WHO Member States through a World Health Assembly resolution in August 2020 [7]. The strategy aims to support countries and relevant stakeholders to translate commitments made in the Moscow Declaration and in the political declaration of the United Nations (UN) high-level meeting on TB into concrete actions [8]. The Sustainable Development Goals (SDG) and End TB Strategy targets set for 2030 cannot be met without intensified research and innovation. Technological breakthroughs are needed by 2025, so that the annual decline in the global TB incidence rate can be accelerated to an average of 17% per year.

## 2. Uzbekistan and Programmatic Management of TB

Uzbekistan is a country in Central Asia with an estimated population of more than 33 million [9]. The country is divided into twelve provinces, the Republic of Karakalpakstan and the metropolitan area of Tashkent, the capital city. TB control activities are coordinated countrywide by the Republican Specialized Scientific Practical Medical Center of Phthisiology and Pulmonology, which is essentially the National TB Programme (NTP). TB diagnosis and treatment are provided free of charge by the NTP. There are no private TB services in the country. All registered TB patients receive treatment in accordance with the national guidelines and protocols, based on the WHO policy recommendations. An established TB laboratory network in the country includes two National Reference Laboratories, several bacteriological (automated cartridge-based nucleic acid amplification tests, line probe assays for detection of resistance to both first- and second-line drugs, liquid and solid culture and phenotypic drug susceptibility testing) and more than 200 sputum smear microscopy laboratories, of which the latter perform direct microscopy of sputum collected in primary healthcare facilities. The mainstay of TB diagnosis in most provinces of Uzbekistan used to be through sputum smear microscopy or X-ray investigations [10], but Xpert MTB/Rif is the primary diagnostic test now in Uzbekistan according to the national diagnostic algorithm.

Uzbekistan is a country with one of the largest burdens of drug-resistant TB (DR-TB) in the WHO European Region. Annually, up to 2500 new patients with multidrug/rifampicin-resistant TB (MDR/RR-TB) are being registered. The drug-resistance survey conducted in 2010–2011 by the Ministry of Health (MoH) of the Republic of Uzbekistan in collaboration with the WHO demonstrated high rates of multidrug-resistant TB (MDR-TB) among new and previously treated patients, 23% and 62% respectively [11], and recent estimates based on the routine drug-resistance surveillance data from 2019 showed that the prevalence of MDR/RR-TB among new patients in Uzbekistan was 12% and 22% among the previously treated patients [1]. Treatment outcomes for TB and MDR/RR-TB cohorts were 92% (2018 cohort) and 61% (2017 cohort), respectively [12]. Treatment success rates in patients with extensively drug-resistant tuberculosis (XDR-TB) are low but are nevertheless substantially higher than the regional average (63% vs. 43%, 2017 cohort).

The average annual decline in the TB incidence rate was 4% during the period 2014–2018, which is lower than the regional average (5%). However, with the average 7% annual decrease in TB mortality, the country is on track to reach the 2020 milestones of the Global End TB strategy and targets articulated in the Regional TB Action Plan.

TB treatment is overseen by the TB dispensaries (outpatient care during the intensive phase and/or continuation phase of treatment) and also at the primary health care level (continuation phase of treatment) for both drug-susceptible and MDR-TB patients in pilot areas. The Global Fund to Fight AIDS, Tuberculosis and Malaria (The Global Fund) provides all first-line anti-TB drugs and, since 2013, has provided all second-line drugs countrywide for treatment of DR-TB. Since 2018, the MoH allocates funds for part procurement of first-line drugs as well. Since 2018, the WHO Country Office in Uzbekistan together with the MoH have started introducing the shorter treatment regimens for MDR/RR-TB patients in some pilot regions of Uzbekistan, including Samarkand and Tashkent city.

Assessment missions organized by the WHO (Green Light Committee, NTP Review) have acknowledged Uzbekistan’s achievements in improving and strengthening TB control in the country. However, the country is experiencing some challenges in the smooth implementation of TB control activities and uptake of the TB research. Like many public health programmes in low- and middle-income countries, the NTP in Uzbekistan “is data rich, but information poor”, implying that much data are generated at the country level but the full potential to use these data to inform improvements in public health is rarely achieved [13]. The area of research is considered by the NTP as one of the important areas that should be strengthened. This could help the MoH and NTP in identifying the potential bottlenecks in the system and the weak areas of the vertical TB control system, which together hinder the scale up of universal access to TB and DR-TB prevention, detection and treatment in Uzbekistan.

The research activities in the TB field are helping NTPs in countries such as Uzbekistan where there is a high burden of MDR-TB) [14] to identify potential bottlenecks and barriers that hinder universal access to services for the prevention, detection and treatment. Research findings provide the evidence base to implement, improve and strengthen sustainable interventions to decrease the burden of TB and DR-TB in Uzbekistan.

## 3. Building Operational Research Capacity in Uzbekistan

Through the leadership of the European Tuberculosis Research Initiative (led by the secretariat at the WHO Regional Office for Europe), and in close collaboration with the Ministry of Health and the NTP, the WHO Country Office in Uzbekistan organized a country-specific Structured Operational Tuberculosis Research Training (SORT-TB). SORT-TB is an adaptation of the training curriculum of the Structured Operational Research and Training Initiative (SORT IT) of the UNICEF/UNDP/World Bank/WHO Special Programme for Research and Training in Tropical Diseases (TDR) for countries of the eastern Europe and central Asian region. The course aims to build the national counterparts’ capacities and skills in operational research in order to fill the current research gaps [13]. The SORT-TB course conducted in Uzbekistan between 2019 and 2020 was co-facilitated by officers from the WHO Regional Office for Europe, the WHO Country Office in Uzbekistan, The International Union Against Tuberculosis and Lung Disease (The Union) in France and individual experts in the area of tuberculosis research.

The call for applications in the course was open for all TB health facilities in the country and interested TB specialists could apply for participation. In total, 23 applications with research proposals were received by the WHO Country Office in Uzbekistan. Through independent review, 13 candidates were selected to attend the course. The minimum requirements were fluent knowledge of the Russian language and data access/availability. Knowledge of English was preferable. The selection was based on the eligibility requirements, as well as on matching of research proposals with national and regional research priorities.

The course was launched on 30 September 2019. In brief, the first and second modules, which were conducted back-to-back, dealt with protocol development, ethics application and data capture, validation and analysis. These two modules were held face-to-face in Samarkand, Uzbekistan. The third module was delayed because of the COVID-19 pandemic and held remotely 14 months later with participants based in Samarkand and external faculty working from home. Despite these challenges, the data were collected, analyzed and the manuscripts were written. After an independent peer review, the research manuscripts are now presented in the current Project Collection issue.

The first group of research manuscripts (Trends, Characteristics and Treatment Outcomes of Patients with Drug-Resistant Tuberculosis in Uzbekistan: 2013–2018; Treatment Outcomes of Isoniazid-Resistant (Rifampicin Susceptible) Tuberculosis Patients in Uzbekistan, 2017–2018; Treatment Compliance of Multidrug Resistant Tuberculosis in Uzbekistan: Does Practice Follow Policy? Scaling Up Molecular Diagnostic Tests for Drug-Resistant Tuberculosis in Uzbekistan from 2012–2019: Are We on the Right Track?; Effectiveness and Safety of a Shorter Treatment Regimen in a Setting with a High Burden of Multidrug-Resistant Tuberculosis; Factors Associated with Unfavourable Treatment Outcomes in Patients with Tuberculosis: a 16-year Cohort Study (2005–2020), Republic of Karakalpakstan, Uzbekistan) looked at different factors associated with case management of DR-TB in Uzbekistan. The study led by Dr. Safaev showed characteristics and outcomes of patients treated for DR-TB from 2013 to 2018 and assessed risk factors for adverse treatment outcomes in those treated in Tashkent city. Particularly, his study found that in Uzbekistan, XDR-TB, male sex, increasing age, previous TB treatment, alcohol abuse and associated comorbidities are factors that increase risk of unfavorable treatment outcomes. The study led by Dr Sayfutdinov found high treatment success among TB patients with “isoniazid resistance but rifampicin susceptible” treated with a 9 month regimen containing levofloxacin throughout the treatment and second-line injectables in the first three months of treatment. The study led by Dr. Usmanova determined whether MDR-TB treatment practices from 2012 to 2018 in Uzbekistan were compliant to national guidelines in terms of regimens prescribed and weight-based drug dosages given, and showed that about 85% of the patients had weight-based drug dosages compliant with national treatment guidelines. The study led by Dr. Yuldashev documented the scale-up of Xpert MTB/RIF assays and line probe assays (LPA) from 2012 to 2019 and looked at how these were associated with increased detection of laboratory-confirmed XDR-TB cases. Dr. Sharaf noted the progress achieved in roll-out of rapid molecular diagnostics in Uzbekistan. The study led by Dr. Trubnikov showed that despite increased doses of TB drugs in the regimen, the short treatment regimens were not associated with excessive drug-toxicity compared with conventional treatment. Dr. Gadoev revealed in his study the main factors associated with unfavourable treatment outcomes in patients with tuberculosis. Factors associated with unfavourable treatment outcomes for the patients who received treatment in Republic of Karakalpakstan, included: increasing age, living in certain parts of the republic, disability, pensioner status, unemployment, being HIV-positive, having pulmonary TB and receiving category II treatment.

The second group of research manuscripts (Diagnostic Procedures, Diagnoses and Treatment Outcomes of Patients with Presumptive Tuberculosis Pleural Effusion in Uzbekistan; Hospitalizations and Treatment Outcomes in Patients with Urogenital Tuberculosis in Tashkent, Uzbekistan, 2016–2018; Characteristics and Treatment Outcomes of Patients with Tuberculosis Receiving Adjunctive Surgery in Uzbekistan) published in the current issue looked at clinical aspects of different types of extra pulmonary TB and surgical interventions during TB treatment. The study led by Dr. Abdugaparov showed that bacteriological confirmation among those diagnosed with pleural effusion was low at 15% and that drug resistance significantly increased the risk of unsuccessful treatment outcome for patients diagnosed with TB pleural effusion. The study led by Dr. Ismatov on length of stay and associated factors among patients with urogenital tuberculosis (UGTB) receiving first-line treatment during 2016–2018 showed high rates of hospitalization and demonstrated that treatment success was not associated with hospitalization endorsing the WHO recommendations in favour of ambulatory models of care. In his study, Dr. Riskiev described the characteristics and treatment outcomes of 101 TB patients receiving adjuvant surgery and concluded that the adjuvant surgical therapy can be an option for TB treatment.

The third group of research manuscripts (Incidence Rate and Risk Factors for Tuberculosis among People Living with HIV: A 2015–2017 Cohort from Tashkent, Uzbekistan; Risk Factors for Unfavorable Treatment Outcomes Among the Human Immunodeficiency Virus Associated Tuberculosis Population in Tashkent City, Uzbekistan: 2013–2017; Universal Access to Xpert MTB/RIF Testing for Diagnosis of Tuberculosis in Uzbekistan: How Well Are We Doing? Adverse Drug Reactions among Children with Tuberculosis in Tashkent, Uzbekistan, 2019) published in the current issue looked at TB/HIV co-infection, TB laboratory issues and adverse drug reactions during TB treatment among children. Dr. Sadirova and colleagues found that antiretroviral therapy (ART) was essential to prevent active TB among people living with HIV (PLHIV), a finding in line with other research studies conducted in different countries. The study led by Dr. Massavirov demonstrated that having extrapulmonary TB, smear-positive laboratory results on admission and co-morbidities of diabetes and hepatitis C were independent risk factors for unfavorable TB treatment outcomes among patients with HIV-associated TB in the country. The study led by Dr. Turaev described the utilization of rapid molecular TB diagnosis (Xpert MTB/RIF and Xpert MTB/RIF Ultra). Furthermore, risk factors for not getting tested with Xpert MTB/RIF included the patient’s age, distance to Xpert MTB/RIF laboratory, diagnostic capacity and the site of the primary health center. The study led by Dr. Abdusalomova illustrated the characteristics associated with adverse drug reactions among children receiving TB treatment and these included treatment using second-line drugs, being treated at the regional hospital, being female and so on.

These studies and their findings now serve as a solid base of evidence to improve TB management activities in Uzbekistan and the training and mentorship of the participants have helped to build and accelerate research capacity in the country. Continuous prioritization, review of TB research gaps at the country level and their alignment with the regional research priorities, outlined in the Regional TB Research Agenda [15], will streamline individual efforts of scientists and research institutions in producing reliable evidence and inform programmatic actions in the management of TB. Joint collaboration of the MoH, the NTP, researchers, scientific institutions, doctors, civil society and patient organizations in research processes is needed to ensure that research benefits serve the purpose of achieving better health outcomes for people with TB in Uzbekistan.
